# Tough decoy targeting of predominant *let*-*7 miRNA* species in adult human hematopoietic cells

**DOI:** 10.1186/s12967-017-1273-x

**Published:** 2017-08-02

**Authors:** Jaira F. de Vasconcellos, Colleen Byrnes, Y. Terry Lee, Joshua M. Allwardt, Megha Kaushal, Antoinette Rabel, Jeffery L. Miller

**Affiliations:** Molecular Genomics and Therapeutics Section, Genetics of Development and Disease Branch, National Institute of Diabetes and Digestive and Kidney Diseases, National Institutes of Health, 10 Center Drive, Building 10, Room 9N311, Bethesda, MD 20892 USA

**Keywords:** *let*-*7*, miRNAs, *let*-*7a*, *let*-*7b*, *Gamma*-*globin*, Fetal hemoglobin, HbF

## Abstract

**Background:**

In humans, the heterochronic cascade composed of the RNA-binding protein LIN28 and its major target, the *let*-*7* family of microRNAs (miRNAs), is highly regulated during human erythroid ontogeny. Additionally, down-regulation of the *let*-*7* miRNAs in cultured adult CD34(+) cells or the over-expression of LIN28 in cultured erythrocytes from pediatric patients with HbSS genotype causes increased levels of fetal hemoglobin (HbF) in the range of 19–40% of the total. Therefore, we hypothesized that focused targeting of individual *let*-*7* miRNA family members would exhibit regulatory effect on HbF expression in human adult erythroblasts.

**Methods:**

The expression levels of mature *let*-*7* family members were measured by RT-qPCR in purified cell populations sorted from peripheral blood. To study the effects of *let*-*7* miRNAs upon globin expression, a lentiviral construct that incorporated the tough decoy (TuD) design to target *let*-*7a* or *let*-*7b* was compared with empty vector controls. Transductions were performed in CD34(+) cells from adult healthy volunteers cultivated ex vivo in erythropoietin-supplemented serum-free media for 21 days. Downstream analyses included RT-qPCR, Western blot and HPLC for the characterization of adult and fetal hemoglobins.

**Results:**

The expression of individual *let*-*7* miRNA family members in adult peripheral blood cell populations demonstrated that *let*-*7a* and *let*-*7b* miRNAs are expressed at much higher levels than the other *let*-*7* family members in purified adult human blood cell subsets with expression being predominantly in reticulocytes. Therefore, we focused this study upon the targeted inhibition of *let*-*7a* and *let*-*7b* with the TuD design to explore its effects upon developmentally-timed erythroid genes. Let-7a-TuD transductions significantly increased *gamma*-*globin* mRNA expression and HbF to an average of 38%. Let-7a-TuD also significantly decreased the mRNA expression of some ontogeny-regulated erythroid genes, namely *CA1* and *GCNT2*. In addition, the erythroid-related transcription factors BCL11A and HMGA2 were down- and up-regulated, respectively, by let-7a-TuD, while ZBTB7A, KLF1 and SOX6 remained unchanged.

**Conclusions:**

Overall, our data demonstrate that *let*-*7* miRNAs are differentially expressed in human hematopoietic cells, and that targeted inhibition of the highly-expressed species of this family is sufficient for developmentally-specific changes in *gamma*-*globin* expression and HbF levels.

**Electronic supplementary material:**

The online version of this article (doi:10.1186/s12967-017-1273-x) contains supplementary material, which is available to authorized users.

## Background

MicroRNAs (miRNAs) are small (mostly 18–21 nucleotides long), non-coding RNAs, highly conserved across evolution and involved in the regulation of messenger RNAs (mRNAs). Intracellular miRNAs cause post-transcriptional repression of multiple mRNAs to which they bind [1]. The *let*-*7* family of miRNAs in humans consists of twelve genes that encode nine mature miRNAs (*let*-*7a*, *let*-*7b*, *let*-*7c*, *let*-*7d*, *let*-*7e*, *let*-*7f*, *let*-*7g*, *let*-*7i* and *miR*-*98*). The expression of mature *let*-*7* miRNAs can be regulated at the transcriptional and post-transcriptional levels, where post-transcriptional repression of *let*-*7* (at both *pri*-*let*-*7* and *pre*-*let*-*7* stages) is mainly mediated by the RNA-binding protein LIN28 [2]. Two human homologs of the *C. elegans lin28* gene were identified and named *LIN28A* and *LIN28B* [3]. Interestingly, LIN28 proteins and their *let*-*7* miRNA targets have several reported functions including regulation of developmental timing [4–7], stem cell pluripotency, and differentiation of skeletal muscle [8, 9].

In humans, reticulocyte levels of *let*-*7* miRNAs increase with the fetal-to-adult developmental transition [10]. *LIN28B* expression is silenced during the same developmental switch. Transgenic increases of LIN28 proteins in adult erythroblasts, which as a consequence down-regulate the *let*-*7* miRNAs, cause the cells to manifest fetal-like features [11, 12]. Augmented expression of *LIN28A/B* also precipitated a rise in fetal hemoglobin (HbF) levels and amelioration of the sickling morphologies of enucleated erythrocytes cultured in vitro from pediatric patients with sickle cell disease (HbSS genotype) [11, 12]. Earlier efforts aimed toward the reduced expression of *let*-*7* by “sponge” targeting of the miRNA family seed region [11] resulted in mild HbF increases compared with LIN28 over-expression in the same cells [11]. Therefore, it remained inconclusive whether suppression of the *let*-*7* family, or targeting of individual *let*-*7* species are sufficient to cause the robust developmentally-specific changes in cellular phenotype that were manifested by LIN28 [11].

Here we investigate the expression levels of the individual *let*-*7* miRNAs in human blood cells, and further explore the role of *let*-7 miRNAs upon ontogeny-related gene expression in the erythroid lineage. Expression of individual *let*-*7* miRNA family members was quantitated in human peripheral blood cell populations, allowing a more focused strategy for reducing *let*-*7* levels in the adult erythroblasts. Finally, lentiviruses designed specifically for *let*-*7a* and *let*-*7b* miRNA targeting were transduced in erythroblasts and explored for their regulation of HbF and other developmentally-regulated genes.

## Methods

### Ethics statement

Written informed consent was obtained from all research subjects prior to participation in this study. Approval for the research protocol and consent documents using primary erythroblasts and peripheral blood samples was granted by the Intramural National Institute of Diabetes and Digestive and Kidney Diseases Institutional Review Board.

### miRBase

Mature *let*-*7* miRNAs sequences were obtained from the miRBase database release 21 (http://mirbase.org). Details of the miRBase database have been previously described [13–17].

### Peripheral blood samples

Peripheral blood cells were isolated using Ficoll-Paque Premium (GE Healthcare, Pittsburgh, PA) following manufacturer’s instructions. Fresh post-ficoll peripheral blood cells were used for cell sorting based on forward and side scatter using the BD FACSAria I flow cytometer (BD Biosciences, San Jose, CA). Lymphocytes and monocytes were sorted from the post-ficoll interface. Neutrophils were obtained from the post-ficoll packed red cells, after lysis with ACK lysing buffer following manufacturer’s protocol (Life Technologies, Grand Island, NY) and sorted based on forward and side scatter. Reticulocytes were obtained after filtration through a Purecell Neonatal High Efficiency Leukocyte Reduction Filter (PALL, Port Washington, NY) of the post-ficoll packed red cells. RNA from lymphocytes, monocytes and neutrophils was extracted using miRNeasy mini kit with QIAzol (Qiagen, Germantown, MD) and RNA from reticulocytes was extracted using Trizol LS following manufacturer’s instructions (Thermo Fisher Scientific, Grand Island, NY).

### Cell culture

Cryopreserved healthy adult human CD34(+) cells were cultured ex vivo in a 3-week serum-free system consisting of three phases (phase I: days 0–7, phase II: days 7–14 and phase III: days 14–21) as previously described [11, 18].

### Recombinant viral transduction

Lentiviral particles with tough decoy (TuD) design [19], constructed to inhibit human *let*-*7a* or *let*-*7b* miRNAs (catalog numbers: HLTUD0001 and HLTUD0007, respectively) and negative vector control (HLTUD001C) were purchased from Sigma Aldrich (St. Louis, MO). A lentivirus shRNA vector to knockdown *BCL11A* (clone TRCN0000033449) and the respective lentiviral control (SHC002V) were also acquired from Sigma Aldrich. On culture day 3 of phase I, CD34(+) cells were transduced with the following lentiviral particles: let-7a-TuD, let-7b-TuD, BCL11A knockdown and each respective negative vector control (MOI of 6). After 24 h, puromycin (Sigma Aldrich) was added to the culture. On culture day 7, cells were transferred to phase II medium containing EPO and cultivated at the conditions previously described without puromycin [18].

### Cell counts and cell morphology analyses

Cell counts were performed throughout the culture period in a Z1 Coulter Particle Counter (Beckman Coulter, Indianapolis, IN) following manufacturer’s instructions. Cell morphology was analyzed with the preparation of cytospins followed by Wright–Giemsa staining. Briefly, cytospins were prepared by centrifugation of the cytoslides using the Shandon Cytospin 4 (Thermo Fisher Scientific) at 1000 rpm for 2 min. Cytoslides were stained with Wright–Giemsa (Sigma-Aldrich, St. Louis, MO) for 50 s followed by two 1-min washes in distilled water.

### Flow cytometry analyses

Erythroid differentiation was assessed with antibodies directed against CD71 and glycophorin A (Invitrogen, Carlsbad, CA) on culture days 14 and 21 using the BD FACSAria I flow cytometer (BD Biosciences) as previously described [20]. Enucleation was quantitated by thiazole orange (TO) staining (Sigma) on culture day 21. Fetal hemoglobin distribution was assessed with antibody directed against fetal hemoglobin (Life Technologies) at culture day 21 as previously described [21].

### Quantitative PCR for mRNAs

Total RNA was isolated using miRNeasy mini kit with QIAzol (Qiagen) following manufacturer’s instructions and complementary DNA (cDNA) was synthesized using SuperScript III reverse transcriptase (Thermo Fisher) following manufacturer’s instructions as previously described [22, 23]. RT-qPCR assays and conditions were performed as previously described [11, 22–25]. Assay-on-Demand Gene Expression Product (Thermo Fisher Scientific/Applied Biosystems) were used as follows: *CA1* (Hs01100176_m1), *GCNT2* (Hs00377334_m1), *BCL11A* (Hs00256254_m1), *HMGA2* (Hs00971724_m1), *ZBTB7A* (Hs00792219_m1), *KLF1* (Hs00610592_m1), *SOX6* (Hs00264525_m1), *LIN28A* (Hs04189307_g1), and *LIN28B (*Hs01013729_m1). Absolute quantification for each target mRNA was determined by comparison with a standard curve that was run in parallel with biological samples as previously described [23]. Reactions were performed in triplicate.

### Quantitative PCR analysis for the *let*-*7* family of miRNAs

Complementary DNA and real-time PCR reaction using Taqman microRNA assay (Applied Biosystems, Grand Island, NY) were performed as previously described [10, 22] for *let*-*7a*, *let*-*7b*, *let*-*7c*, *let*-*7d*, *let*-*7e*, *let*-*7f*, *let*-*7* *g*, *let*-*7i* and *miR*-*98*. Absolute quantification for each target miRNA was determined by comparison with a standard curve that was run in parallel with biological samples as previously described [22]. Standard curves were prepared on the basis of the synthetic targeted mature miRNA oligonucleotide of known concentration (at least five 1:10 serial dilutions) as previously described [22]. Reactions were performed in triplicate. A representative standard curve and its correspondent amplification plot for each *let*-*7* miRNA family member is shown in Additional file 1.

### Western blot analyses

Nuclear and cytoplasmic extracts from culture day 14 erythroblasts were prepared using the NE-PER Nuclear and Cytoplasmic Extraction kit (Pierce Biotechnology, Rockford, IL) as previously described [11]. Western blot protocols and conditions were performed as previously described [11]. Blots were probed with antibodies against CA1 (Abcam, Cambridge, MA), GCNT2 (Santa Cruz Biotechnology, Dallas, TX), BCL11A (Abcam), HMGA2 (GeneTex, Irvine, CA), ZBTB7A (Abcam), KLF1 (Abcam) and SOX6 (Santa Cruz Biotechnology). Histone H3, Lamin B1 or Beta-Actin (all from Abcam) were used as loading controls.

### Colony formation assay

CD34(+) cells from three independent donors were transduced with *let*-*7a* tough decoy vector (catalog number: HLTUD0001, Sigma) or negative vector control (catalog number: HLTUD001C, Sigma) overnight and then mixed in MethoCult H4034 Optimum media (Stem Cell Technologies, Vancouver, Canada) supplemented with puromycin for colony formation assay with duplicate wells following manufacturer’s protocol as previously described [18]. Colonies of erythroid progenitors (BFU-E and CFU-E), granulocyte–macrophage progenitors (CFU-GM, CFU-G and CFU-M) and multipotential granulocyte, erythroid, macrophage, megakaryocyte progenitors (CFU-GEMM) were counted for each donor and condition on culture day 14.

### HPLC for adult and fetal hemoglobins

Samples for HPLC analysis were prepared and analyzed as previously described [23, 26].

### Statistical analysis

Replicates are expressed as mean ± SD values and significance was calculated by two-tailed Student’s t-test.

## Results

### Reticulocytes contain higher total levels of mature *let*-*7* miRNAs than other peripheral blood cell populations

**Fig. 1:**
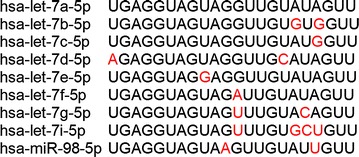
The human *let*-*7* family of miRNAs. The nucleotide differences of the human mature *let*-*7* family members in comparison to the human mature *let*-*7a* miRNA are marked in red font. Sequences were obtained using miRBase (http://mirbase.org/), Release 21

**Fig. 2:**
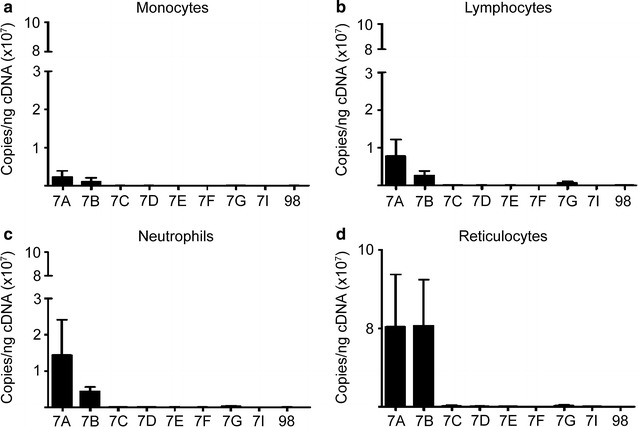
*Let*-*7a* and *let*-*7b* are predominant miRNAs among the *let*-*7* family members in peripheral blood cells. Levels of mature *let*-*7* miRNAs in **a** monocytes (n = 4), **b** lymphocytes (n = 4), **c** neutrophils (n = 4) and **d** reticulocytes (n = 5). Samples were analyzed by RT-qPCR quantitation of copy number *per* nanogram of complementary DNA (cDNA) (copies/ng cDNA). Mean value ± SD of independent donors for each condition

The mature sequences of *let*-*7* miRNA family members are defined and well-conserved across multiple species. As shown in Additional file 2, *let*-*7a* is the most well-conserved family member across evolution. The alignment and nucleotide differences of the human mature *let*-*7* family members in comparison to the human mature *let*-*7a* miRNA is shown in Fig. 1 (sequences were obtained on miRBase, http://mirbase.org/, Release 21). Following the in silico comparison of the *let*-*7* miRNAs sequences, we explored the expression levels of mature *let*-*7a* and the related miRNA family members in purified mononuclear cell populations and reticulocytes from peripheral blood. Interestingly, reticulocytes have higher levels of total *let*-*7* miRNAs compared to monocytes, lymphocytes, and neutrophils (Fig. 2; monocytes: 3.5E+06 ± 2.7E+06 copies/ng; lymphocytes: 1.1E+07 ± 6.2E+06 copies/ng; neutrophils: 2.0E+07 ± 1.1E+07 copies/ng and reticulocytes: 1.7E+08 ± 1.0E+08 copies/ng). Unexpectedly, among the individual family members, *let*-*7a* and *let*-*7b* were identified as the predominant members of the *let*-*7* family in peripheral blood cell populations. Hence, we became more interested in the effects of focused reductions of these highly-expressed species, *let*-*7a* and *let*-*7b*, in erythroblasts.

### Studies of cell proliferation and differentiation showed decreased enucleation upon *let*-*7* miRNAs focused inhibition in adult erythroblasts cultivated ex vivo

**Fig. 3:**
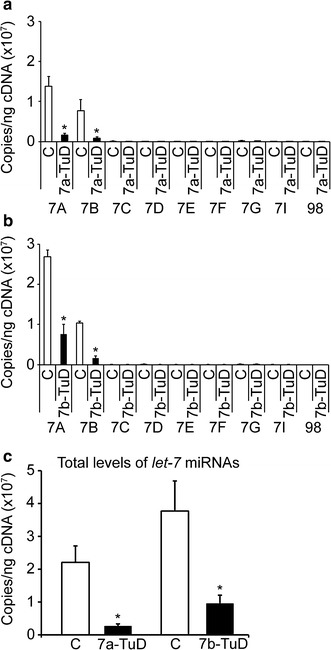
*Let*-*7* miRNAs focused inhibition by TuD is accompanied by the reduction of mature *let*-*7* levels. Quantitation of copy number per nanogram cDNA (copies/ng cDNA) by RT-qPCR in **a** let-7a-TuD (n = 5) and **b** let-7b-TuD (n = 4), both compared to control transductions. **c** Total levels of mature *let*-*7* miRNAs in let-7a-TuD and let-7b-TuD treatments compared to control. *Open bars* represent control and *black bars* represent let-7a-TuD or let-7b-TuD. Mean value ± SD of independent donors for each condition. p values were calculated for comparison of control versus *let*-*7a*, *let*-*7b* or the total levels of *let*-*7* miRNAs using two-tailed Student’s t-test. TuD, tough decoy design; C, control (negative control vector) transduction; 7a-TuD, *let*-*7a* tough decoy design; 7b-TuD, *let*-*7b* tough decoy design. *p < 0.05

To investigate the inhibition of *let*-*7a* and *let*-*7b* miRNAs, lentiviral constructs that incorporated the Tough Decoy (TuD) design targeting *let*-*7a* or *let*-*7b* were compared with control vector. Transductions were performed in CD34(+) cells from adult healthy volunteers cultivated ex vivo in erythropoietin-supplemented serum-free media for 21 days. The effects of miRNA inhibition by TuD vectors has been shown to be accompanied by a decrease of target miRNA levels [27], as a result of target-miRNA sequestration [27, 28] and degradation, the latter via the ‘tailing and trimming’ pathway [28]. As shown in Fig. 3a, b, inhibition of *let*-*7* miRNAs by TuD caused reduction in the levels of *let*-*7* species as assessed by RT-qPCR at culture day 14 (*let*-*7a* RT-qPCR: control: 1.4E+07 ± 2.4E+06 copies/ng, let-7a-TuD: 1.6E+06 ± 4.6E+05 copies/ng, p = 0.0003; *let*-*7b* RT-qPCR: control: 1.0E+07 ± 4.9E+05, let-7b-TuD: 1.6E+06 ± 5.1E+05, p = 0.00002). Importantly, both let-7a-TuD and let-7b-TuD reduced the expression of both *let*-*7a* and *let*-*7b* miRNAs, demonstrating a more focused, but not exclusive targeting of miRNA by this technology. When the total levels of *let*-*7* miRNAs were compared between let-7a-TuD and let-7b-TuD designs, the let-7a-TuD vector was more efficient in reducing the total levels of *let*-*7* miRNAs compared to let-7b-TuD (Fig. 3c; 88% versus 75% suppression of total *let*-*7* miRNA levels after let-7a-TuD and let-7b-TuD transductions, respectively). Since the data suggested that let-7a-TuD resulted in a greater reduction in total levels of *let*-*7* miRNAs compared to let-7b-TuD, the let-7a-TuD vector was utilized for subsequent studies.

**Fig. 4:**
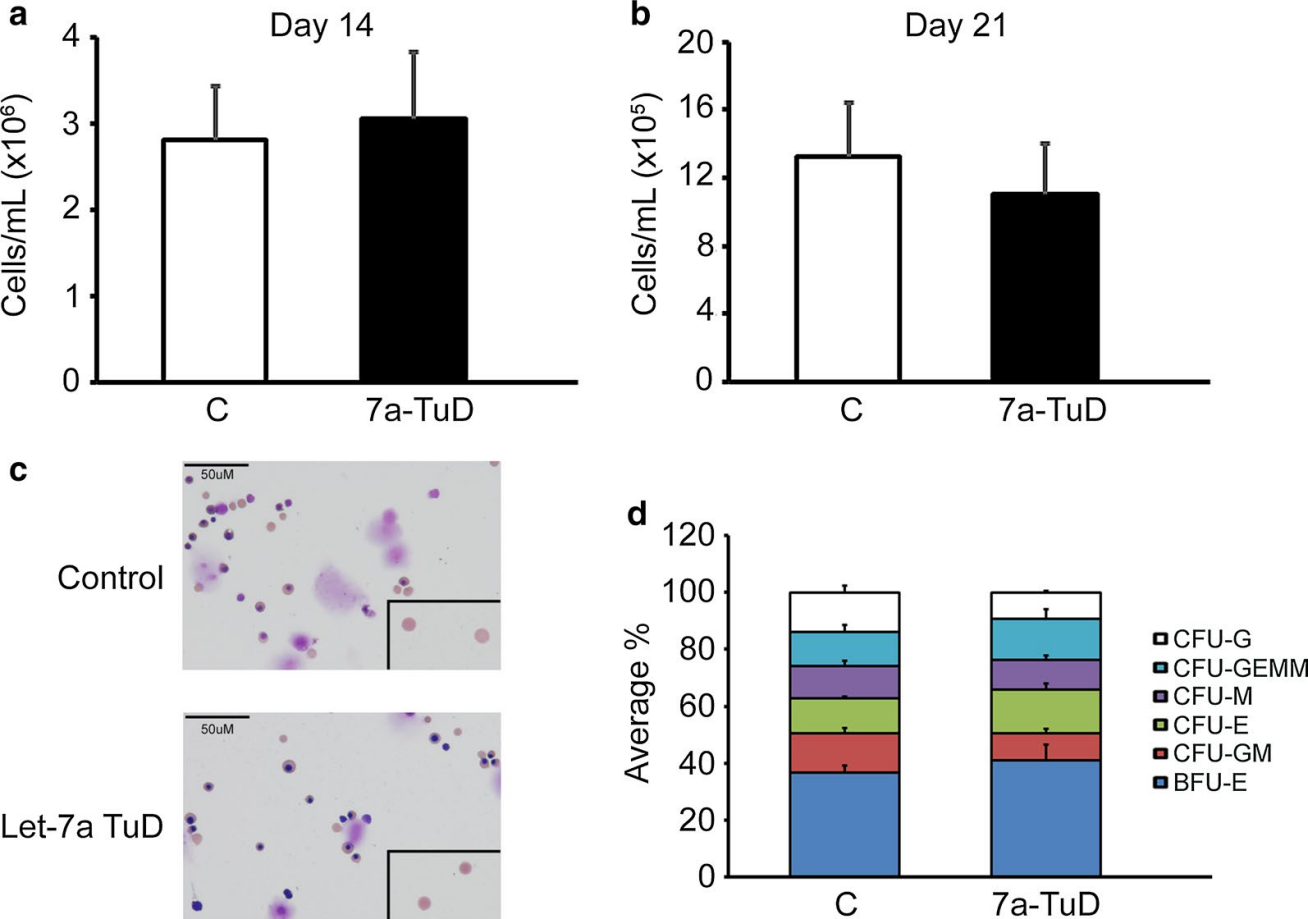
*Let*-*7* miRNAs focused inhibition by TuD demonstrates comparable levels of cell proliferation and equivalent cell morphology compared to control transductions. Cell proliferation was assessed by cell counts (cells/mL) performed at cultures **a** day 14 and **b** day 21. *Open bars* represent control and *black bars* represent let-7a-TuD. Mean value ± SD of five independent donors for each condition. Cell morphology was analyzed at culture day 21 with the preparation of cytospins followed by Wright–Giemsa staining. Representative images are shown from **c** control transduction and let-7a-TuD with enucleated cells (inset). **d** Colony formation assay was performed in control transduction and let-7a-TuD at culture day 14. Cells were transduced with lentivirus particles and cultured in semi-solid methylcellulose medium supplemented with puromycin. Average colony counts were obtained from duplicate wells for each condition from three independent donors. Average percentage of each colony type is shown as separate *colors* (see *color* key on the* right* of the *bar* graph). C, control transduction; let-7a-TuD, let-7a tough decoy design

Cell proliferation and erythroblast differentiation were compared between control transductions and let-7a-TuD. Similar cell counts were observed between let-7a-TuD versus control transductions at culture days 14 and 21 of differentiation (Day 14: control: 2.81E+06 ± 6.24E+05 cells/mL, let-7a-TuD: 3.06E+06 ± 7.77E+05 cells/mL, p = 0.31, Fig. 4a; Day 21: control: 1.32E+06 ± 3.15E+05 cells/mL, let-7a-TuD: 1.10E+06 ± 2.98E+05 cells/mL, p = 0.18, Fig. 4b). In addition, let-7a-TuD cells were morphologically comparable to control transductions at culture day 21 (Fig. 4c). To investigate the effects of the let-7a-TuD vector on the survival and ability of the CD34(+) cells to grow into colonies, a colony formation assay was performed. As shown in Fig. 4d, no differences were observed on the number of puromycin-resistant colonies of BFU-E, CFU-GM, CFU-E, CFU-M, CFU-GEMM and CFU-G progenitors on let-7a-TuD samples compared to control transductions.

**Fig. 5:**
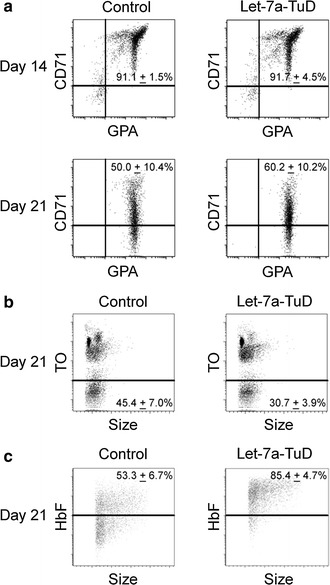
Focused inhibition of *let*-*7* miRNAs reduces in vitro erythroblast enucleation and results in pancellular distribution of HbF. Representative flow dot plots of **a** let-7a-TuD and control transduction at culture days 14 and 21 stained with anti-transferrin receptor (CD71) and anti-glycophorin A (GPA) antibodies. Culture day 21 cells were also assessed for **b** enucleation by thiazole orange (TO) staining and **c** fetal hemoglobin distribution by fetal hemoglobin (HbF) staining for let-7a-TuD and control transduction. Mean value ± SD of five independent donors for CD71, GPA and thiazole orange stains analyses, and of three independent donors for HbF stain. Control, control transduction; let-7a-TuD, let-7a tough decoy design

Flow cytometry analysis of transferrin receptor (CD71) and glycophorin A (GPA) were performed at culture days 14 and 21. As shown in Fig. 5a and Additional file 3A–D, erythroblast differentiation (CD71 and GPA) was not affected by let-7a-TuD. Thiazole orange staining on culture day 21 showed that both let-7a-TuD and control transductions achieved enucleation, however, there was a significant decrease in the percent of enucleated cells in let-7a-TuD compared to control transductions (control: 45.4 ± 7.0%, let-7a-TuD: 30.7 ± 3.9%, p = 0.01; Fig. 5b and Additional file 3E). Finally, HbF distribution was assessed by HbF staining at culture day 21. As shown in Fig. 5c, pancellular expression of HbF was observed upon let-7a-TuD compared to control transductions (control: 53.3 ± 6.7, let-7a-TuD: 85.4 ± 4.7, p = 0.02, Additional file 3F).

### Let-7a-TuD increases *gamma*-*globin* mRNA and fetal hemoglobin levels in cultured adult erythroblasts

**Fig. 6:**
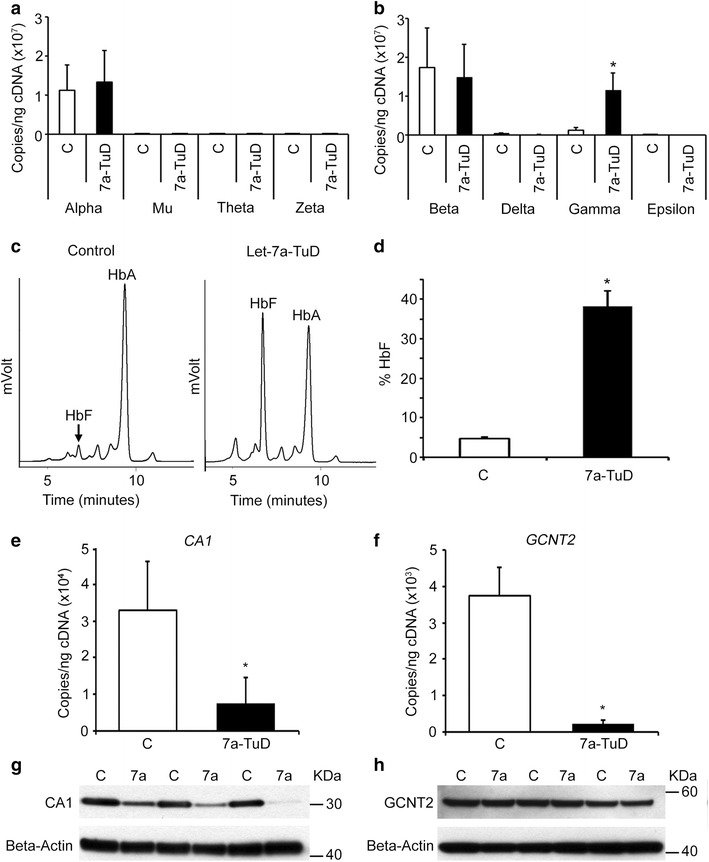
Focused inhibition of *let*-*7* miRNAs regulates *gamma*-*globin* and HbF levels in human erythroblasts ex vivo. Let-7a-TuD and control transductions were investigated for **a** *alpha*-, *mu*-, *theta*- and *zeta*-*globins*, and **b** *beta*-, *delta*-, *gamma*- and *epsilon*-*globins*. RT-qPCR analyses were performed at culture day 14. **c** Representative HPLC tracings. HbF and HbA peaks are labeled on each graph (*y-axis*, mVolts;* x-axis*, elution time in minutes). **d** HPLC analysis of hemoglobin from let-7a-TuD compared to control transductions. HPLC samples were collected at culture day 21. Let-7a-TuD effects in **e** *CA1* and **f** *GCNT2* mRNA levels. RT-qPCR analyses were performed at culture day 14. *Open bars* represent control and *black bars* represent let-7a-TuD. Mean value ± SD of five independent donors for each condition. p values were calculated using two-tailed Student’s t-test. Western blot analyses of **g** CA1 and **h** GCNT2 in the cytoplasmic extracts of three independent donors at culture day 14 upon let-7a-TuD compared to control transductions. Blots were probed with anti-CA1 or anti-GCNT2 antibodies as indicated. Beta-Actin was used as loading control. C, control (negative control vector) transduction; 7a-TuD or 7a, let-7a tough decoy design. *p < 0.05

To further characterize the effects of cultured adult erythroblasts, RT-qPCR analysis of the globin genes was performed at culture day 14. No major differences were observed in *alpha*-, *mu*-, *theta*-, *zeta*-, *beta*-, *delta*- and *epsilon*-*globin* mRNA levels among let-7a-TuD samples compared to control transductions (Fig. 6a, b). However, the *gamma*-*globin* mRNA expression level was significantly increased in let-7a-TuD (control: 1.2E+06 ± 6.8E+05 copies/ng, let-7a-TuD: 1.1E+07 ± 4.5E+06 copies/ng, p = 0.004; Fig. 6b). In addition, hemoglobin profiles (HPLC) were generated at culture day 21, showing a robust increase in HbF levels upon treatment with the TuD lentiviral vector (Fig. 6c, d; control: 4.7 ± 0.6%, let-7a-TuD: 38.2 ± 3.8%, p = 0.00003). For comparison purposes, HPLC analysis of let-7b-TuD was performed and demonstrated that let-7b-TuD caused less pronounced changes in the HbF levels, reaching 29.7 ± 4.5% compared to control transductions at 4.1 ± 0.9% in matched cultures.

### *Let*-*7* miRNAs inhibition by TuD demonstrates additional developmentally-specific gene regulation in adult erythroblasts

The fetal-to-adult transition in humans is accompanied by increased expression in the levels of carbonic anhydrase I (*CA1*), which after globin is the second most abundant protein in adult erythroid cells [29]. In addition, increased expression in the levels of glucosaminyl (N-acetyl) transferase 2 (*GCNT2*) during the fetal-to-adult transition catalyzes the expression of adult blood group I antigen in erythrocytes [30, 31]. To investigate whether let-7a-TuD transduced cells would have effects on these erythroid-relevant developmentally regulated genes, let-7a-TuD cells and control transductions were investigated for the expression of CA1 and GCNT2 by RT-qPCR and Western blot. Interestingly, both *CA1* and *GCNT2* were significantly down-regulated at the mRNA level in let-7a-TuD samples compared to controls *(CA1*: control: 3.3.E+04 ± 1.3.E+04 copies/ng; let-7a-TuD: 7.5.E+03 ± 6.9.E+03 copies/ng; p = 0.005; *GCNT2*: control: 3.8.E+03 ± 7.7.E+02 copies/ng; let-7a-TuD: 2.2.E+02 ± 9.4.E+01; p = 0.002; Fig. 6e, f). However, at the protein level, only CA1 was down-regulated in let-7a-TuD samples compared to controls, while GCNT2 remained unchanged (Fig. 6g, h). Importantly, *LIN28A* and *LIN28B* mRNA transcripts remained at background levels and below the detection limits, respectively, in both let-7a-TuD and control transductions.

**Fig. 7:**
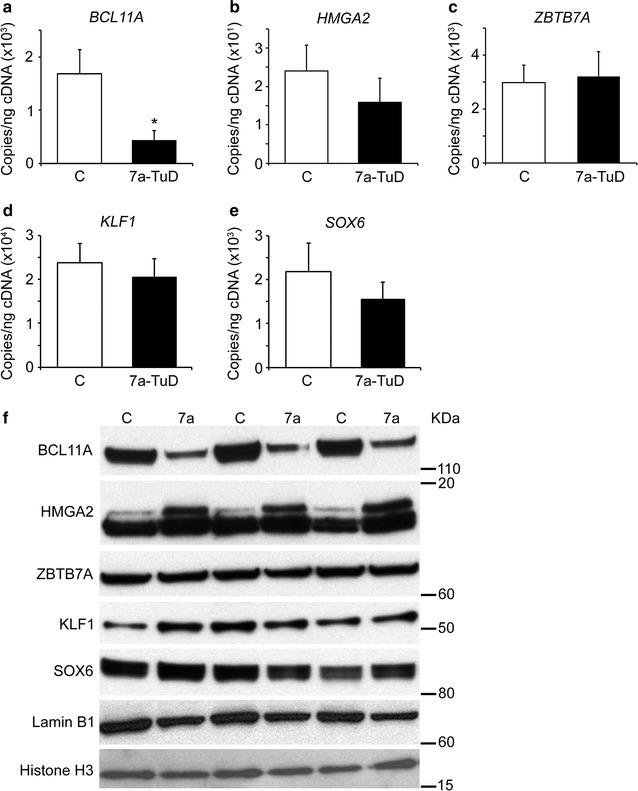
*Let*-*7* miRNAs focused inhibition modulates the expression levels of BCL11A and HMGA2. Let-7a-TuD effects in **a** *BCL11A*, **b** *HMGA2*, **c** *ZBTB7A*, **d** *KLF1* and **e** *SOX6* mRNA levels. RT-qPCR analyses were performed at culture day 14. *Open bars* represent control and *black bars* represent let-7a-TuD. Mean value ± SD of at least four independent donors for each condition. p values were calculated using two-tailed Student’s t-test. **f** Western blot analyses of BCL11A, HMGA2, ZBTB7A, KLF1 and SOX6 in the nuclear extracts of three independent donors at culture day 14 upon let-7a-TuD compared to control transductions. Blots were probed with anti-BCL11A, anti-HMGA2, anti-ZBTB7A, anti-KLF1 or anti-SOX6 antibodies as indicated. Lamin B1 and Histone H3 were used as loading controls. C, control (negative control vector) transduction; 7a-TuD or 7a, let-7a tough decoy design. *p < 0.05

Additionally, the erythroid-related genes (*BCL11A*, *HMGA2*, *ZBTB7A*, *KLF1* and *SOX6*) [22, 32–37] were studied for comparison in let-7a-TuD and control transductions. Interestingly, while *BCL11A* mRNA expression levels were significantly reduced in let-7a-TuD samples compared to control transductions (control: 1.7E+03 ± 4.5E+02 copies/ng; let-7a-TuD: 4.3E+02 ± 1.8E+02 copies/ng; p = 0.003), no significant differences were observed in *HMGA2*, *ZBTB7A*, *KLF1* and *SOX6* transcripts (Fig. 7a–e). However, marked reduction in the protein level of BCL11A was observed after let-7a-TuD (Fig. 7f), while HMGA2 showed a double-band pattern previously reported [38] with a marked increase in the intensity of the upper band after let-7a-TuD (Fig. 7f). Of note, ZBTB7A, KLF1, and SOX6 protein levels demonstrated no consistent change with let-7a-TuD.

### Direct knockdown of *BCL11A* does not significantly affect the *let*-*7* family of miRNAs

**Fig. 8:**
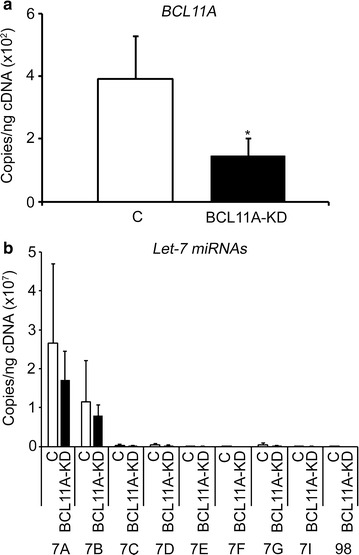
BCL11A does not regulate the *let*-*7* family of miRNAs. **a** *BCL11A* knockdown (BCL11A-KD) was confirmed by RT-qPCR. **b** BCL11A-KD effects in the expression levels of the *let*-*7* family of miRNAs. RT-qPCRs were performed at culture day 14. *Open bars* represent control and *black bars* represent BCL11A-KD. Mean value ± SD of three independent donors for each condition. p value was calculated using two-tailed Student’s t-test. C, control (negative control vector) transduction; BCL11A-KD, *BCL11A* knockdown. *p < 0.05

Finally, to determine whether direct down-regulation of *BCL11A* would affect the *let*-*7* family of miRNAs, primary CD34(+) cells were transduced in this experimental system with a lentivirus shRNA vector to knockdown *BCL11A* as well as the lentiviral vector matched control for comparison. *BCL11A* knockdown (BCL11A-KD) was confirmed by RT-qPCR (Fig. 8a). Interestingly, BCL11A-KD did not significantly affect the *let*-*7* family of miRNAs (Fig. 8b), which suggests that the *let*-*7* miRNAs are upstream regulators of BCL11A.

## Discussion

In this study, we show that the *let*-*7* family of miRNAs is differentially expressed in purified adult human blood, and that *let*-*7a* and *let*-*7b* are the predominantly expressed family members in the analyzed peripheral blood cell populations, including reticulocytes. Focused suppression of *let*-*7a* and *let*-*7b* miRNAs with a miRNA Tough Decoy approach in erythroblasts was sufficient to cause robust changes in several developmentally-specific erythroblast genes including increases in *gamma*-*globin* mRNA expression and HbF to reach mean levels around 35–40% of the total hemoglobin produced. As such, these data confirm a functional role for erythroblast *let*-*7* miRNAs in globin gene regulation and suggest that targeted reductions of the predominant *let*-*7s* should be further explored for application in patients with sickle cell disease and beta-hemoglobin disorders.

The first description of small-RNAs as regulators of developmental timing events was observed in studies of *C. elegans*, when *lin*-*4* and, subsequently *let*-*7* (from the initial denomination *lethal*-*7*) [39–41] were identified. The mature *let*-*7* miRNAs sequence and its function as a heterochronic regulator are highly conserved across evolution [42], and *let*-*7a* is the most well-conserved *let*-*7* family member. Mature *let*-*7a* originated from three different genomic loci (*let*-*7a*-*1*, *let*-*7a*-*2* and *let*-*7a*-*3*) and mature *let*-*7f* originated from miRNA precursors of two distinct genomic locations (*let*-*7f*-*1* and *let*-*7f*-*2*), while all other family members are originated from one precursor miRNA sequence.

Also of interest, we observed that *let*-*7a* and *let*-*7b* are the major species detected by RT-qPCR in all peripheral blood cell populations analyzed. As reported previously, array-based analyses of the *let*-*7* miRNAs demonstrated similarly high levels of each *let*-*7* family member in human adult reticulocytes rather than the predominance of *let*-*7a* and *let*-*7b* [10]. The differences between these microarrays *versus* RT-qPCR results may be due to a higher level of cross-reactivity in *let*-*7* miRNAs array-based detection [43].

Here we aimed our gene transduction studies to suppress the two most prevalent members of the *let*-*7* miRNA family, *let*-*7a* and *let*-*7b.* Importantly, the high similarity among the mature *let*-*7* miRNA sequences prevented the exclusive targeting by tough decoy (TuD) lentiviral designs. While TuD inhibitors are able to provide a more focused inhibition of miRNAs than other strategies [27, 28], similar non-specificity of TuD constructs for targeted species in the same miRNA family was previously described [19]. These results support the notion that targeted *let*-*7* inhibition is a robust approach towards the manipulation of HbF levels in adult erythroblasts. Genomic targeting of individual *let*-*7* species, perhaps with short palindromic repeat technologies, may be useful for determination of individual *let*-*7* family member effects upon HbF.

Interestingly, the magnitude of total *let*-*7* suppression was proportional to the increase in *gamma*-*globin* mRNA and HbF in studies reported to date. It is known that LIN28 proteins regulate *let*-*7* biogenesis and that *let*-*7* miRNAs regulate *LIN28* levels by binding to its 3′ untranslated region in a double negative feedback loop [44]. The absence of increased *LIN28A* or *LIN28B* mRNA transcripts after *let*-*7* suppression suggests that the *LIN28* genes are transcriptionally silent rather than post-transcriptionally degraded by *let*-*7* in the adult cells. Our study also provides the first evidence that the *let*-*7* effects extend beyond globin gene regulation to include other markers of the fetal-to-adult switch in the erythroid lineage, namely *CA1* and *GCNT2*. Interestingly, reduced levels were observed only at the protein levels of CA1, while GCNT2 levels remained unchanged. The biological significance of this finding will require further investigation.

The erythroid-related genes *BCL11A*, *HMGA2*, *ZBTB7A*, *KLF1* and *SOX6* were investigated upon *let*-*7* suppression. The B-cell CLL/lymphoma 11A (*BCL11A*) is a zinc-finger transcription factor known to regulate *gamma*-*globin* and HbF levels in human erythroid cells [32] as well as to rescue the sickle cell disease phenotype in a murine model through the activation of HbF [33]. The High Mobility Group AT-hook 2 (*HMGA2*) is known as an architectural transcription factor and a target of the *let*-*7* miRNAs [34] that has been recently reported to regulate *gamma*-*globin* mRNA and moderately increase HbF levels in human adult erythroblasts in vitro [22]. The Leukemia/Lymphoma-Related Factor (LRF) encoded by the Zinc Finger and BTB Domain Containing 7A (*ZBTB7A*) gene is also a zinc-finger transcription factor shown to cause robust increases in the HbF levels in human cultured erythroblasts [35]. The Kruppel like factor 1 (*KLF1*) is an erythroid-specific transcription factor known to regulate the expression of several erythroid genes including *BCL11A* [36]. Finally, the SRY-box 6 (*SOX6*) is a transcription factor that contains a conserved DNA-binding domain and was demonstrated to physically interact and co-occupy the human beta-globin cluster with BCL11A and other transcription factors such as GATA1 [37]. Interestingly, marked modulations were observed only at BCL11A (down-regulation at both the mRNA and protein levels) and HMGA2 (up-regulation at the protein level).

Overall, we interpret our data as demonstrating that reduction of *let*-*7*, in the absence of other potential LIN28 effects, is a main driver of these developmentally-regulated genes in erythroblasts. While LIN28 effects upon the expression of *BCL11A* have been inconsistent in prior studies [11, 12], this study shows that robust *let*-*7* reduction is sufficient to reduce BCL11A as well as to increase HMGA2 for increased *gamma*-*globin* transcription. Future studies should be aimed toward understanding how this well-conserved miRNA family is able to regulate erythroid gene activity associated with the fetal-to-adult transition in humans. Since *let*-*7* has a more generic role in timing worm development, such studies may ultimately demonstrate how the *let*-*7* developmental clock circuit functionally evolved in human tissues.

## Conclusions

The *let*-*7* family of miRNAs is differentially expressed among monocytes, lymphocytes, neutrophils, and reticulocytes from adult human blood and higher expression levels of the *let*-*7* miRNAs were observed in reticulocytes. Remarkably, *let*-*7a* and *let*-*7b* are predominantly expressed species in all peripheral blood cell populations analyzed. Also of interest, targeted reduction of *let*-*7a* was more efficient than *let*-*7b*, and focused targeting of *let*-*7a* in erythroblasts is sufficient to cause robust increases in *gamma*-*globin* mRNA expression and HbF to mean levels around 38% of the total hemoglobin produced. Targeting of individual *let*-*7* genes or additional RNA transcripts from the *let*-*7* cascade may be useful for therapeutic induction of HbF levels in patients with sickle cell disease or other beta-hemoglobinopathies.

## Additional files

**Additional file 1.** Representative standard curve and amplification plot from each *let-7* family member RT-qPCR. Standard curve and amplification plot from **(A)** *let-7a*, **(B)** *let-7b*, **(C)** *let-7c*, **(D)** *let-7d*, **(E)** *let-7e*, **(F)** *let-7f*, **(G)** *let-7g*, **(H)** *let-7i* and **(I)** *miR-98.* RT-qPCR quantitation of copy number *per* nanogram of complementary DNA (cDNA) (copies/ng cDNA). Ct = cycle threshold.

**Additional file 2.** Evolutionary conservation of the *let-7* family of miRNAs from representative species progressing from the worm to human. Mature *let-7* sequences from each species compared to the corresponding human sequence. Mature *let-7* sequences from all species were obtained from the miRBase database release 21 (http://mirbase.org). Some members of the family may be missing due to incomplete sequencing, rather than their absence from a species.

**Additional file 3.** Quantitative analysis of the flow cytometric results measured by fluorescence-activated cell analysis of control transduction and let-7a-TuD. **(A)** Percentage of CD71(+) and GPA(+) cells or **(B)** CD71(+) and GPA(-) cells at culture day 14. **(C)** Percentage of CD71(+) and GPA(+) cells or **(D)** CD71(-) and GPA(+) cells at culture day 21. **(E)** Percentage of thiazole orange negative cells (enucleated) at culture day 21. **(F)** Percentage of fetal hemoglobin positive cells at culture day 21. Open bars represent control and black bars represent let-7a-TuD. Mean value ± SD of five independent donors for CD71, GPA and thiazole orange stains. Mean value ± SD of three independent donors for HbF stain. P values were calculated using two-tailed Student’s t-test. CD71 = anti-transferrin receptor; GPA = anti-glycophorin A; TO = thiazole orange; HbF = fetal hemoglobin; C = control (negative control vector) transduction; 7a-TuD = let-7a tough decoy design. *p<0.05.

## Abbreviations

BCL11A: B-cell CLL/lymphoma 11A gene
BCL11A-KD: BCL11A knockdown
CA1: carbonic anhydrase I gene
cDNA: complementary DNA
CD71: transferrin receptor
Copies/ng cDNA: copy number per nanogram cDNA
GCNT2: glucosaminyl (N-acetyl) transferase 2 gene
GPA: glycophorin A
HbF: fetal hemoglobin
HMGA2: high mobility group AT-hook 2 gene
HPLC: high-performance liquid chromatography
KLF1: Kruppel like factor 1 gene
Let-7a-TuD: let-7a tough decoy design
Let-7b-TuD: let-7b tough decoy design
LIN28A: lin-28 homolog A gene
LIN28B: lin-28 homolog B gene
miRNAs: microRNAs
mRNAs: messenger RNAs
RT-qPCR: quantitative reverse-transcriptase polymerase chain reaction
SOX6: SRY-box 6 gene
TO: thiazole orange
TuD: tough decoy
ZBTB7A: zinc finger and BTB domain containing 7A gene
