# Supplementary figures and images for: Tough decoy targeting of predominant let-7 miRNA species in adult human hematopoietic cells
Source: J Transl Med. 2017 Aug 2;15:169. doi: 10.1186/s12967-017-1273-x (PMC5541688; doi:10.1186/s12967-017-1273-x)

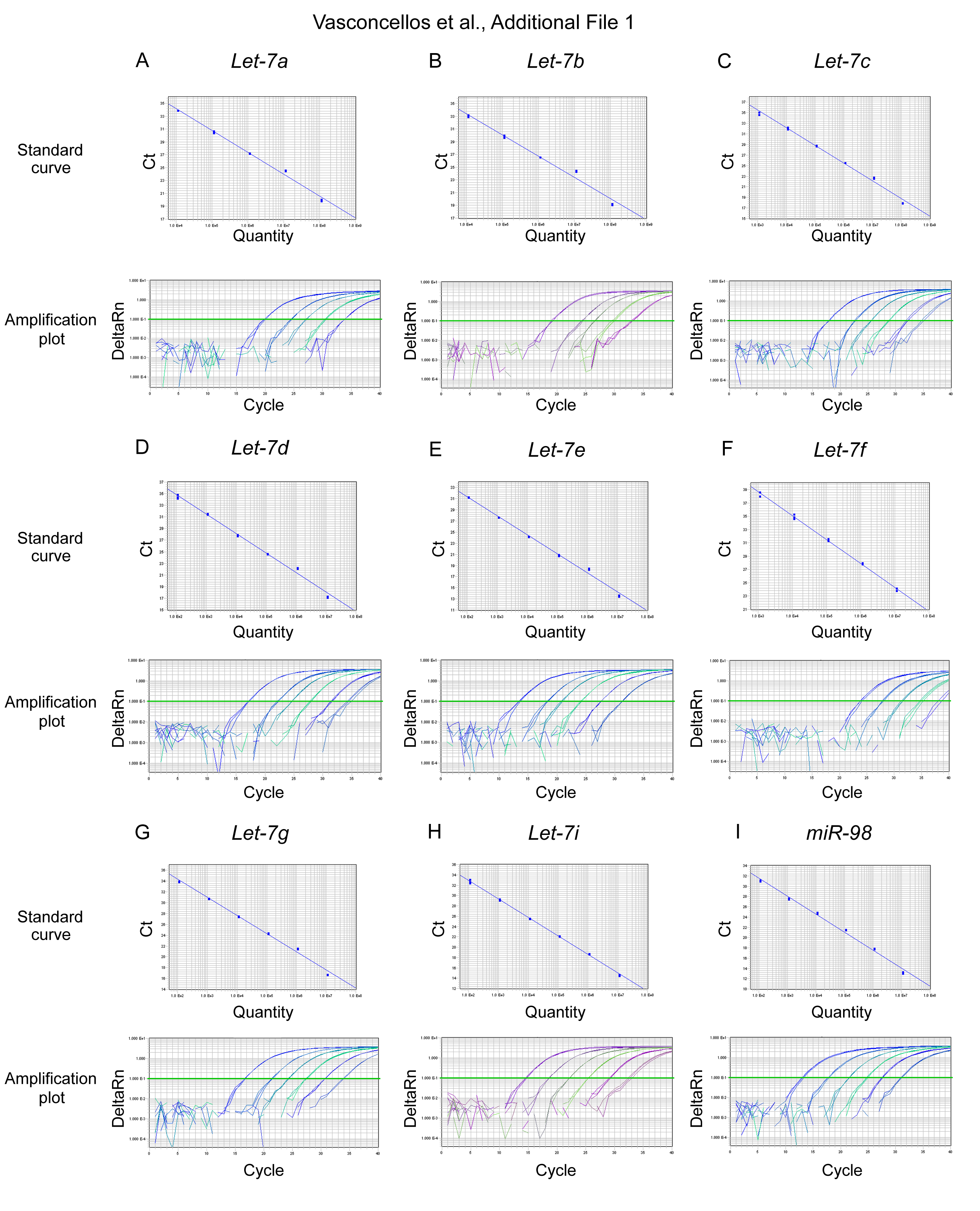

Supplement: Supplementary file 1 — Additional file 1. Representative standard curve and amplification plot from each let-7 family member RT-qPCR. Standard curve and amplification plot from (A) let-7a, (B) let-7b, (C) let-7c, (D) let-7d, (E) let-7e, (F) let-7f, (G) let-7g, (H) let-7i and (I) miR-98. RT-qPCR quantitation of copy number per nanogram of complementary DNA (cDNA) (copies/ng cDNA). Ct = cycle threshold. [file 12967_2017_1273_MOESM1_ESM.tif]

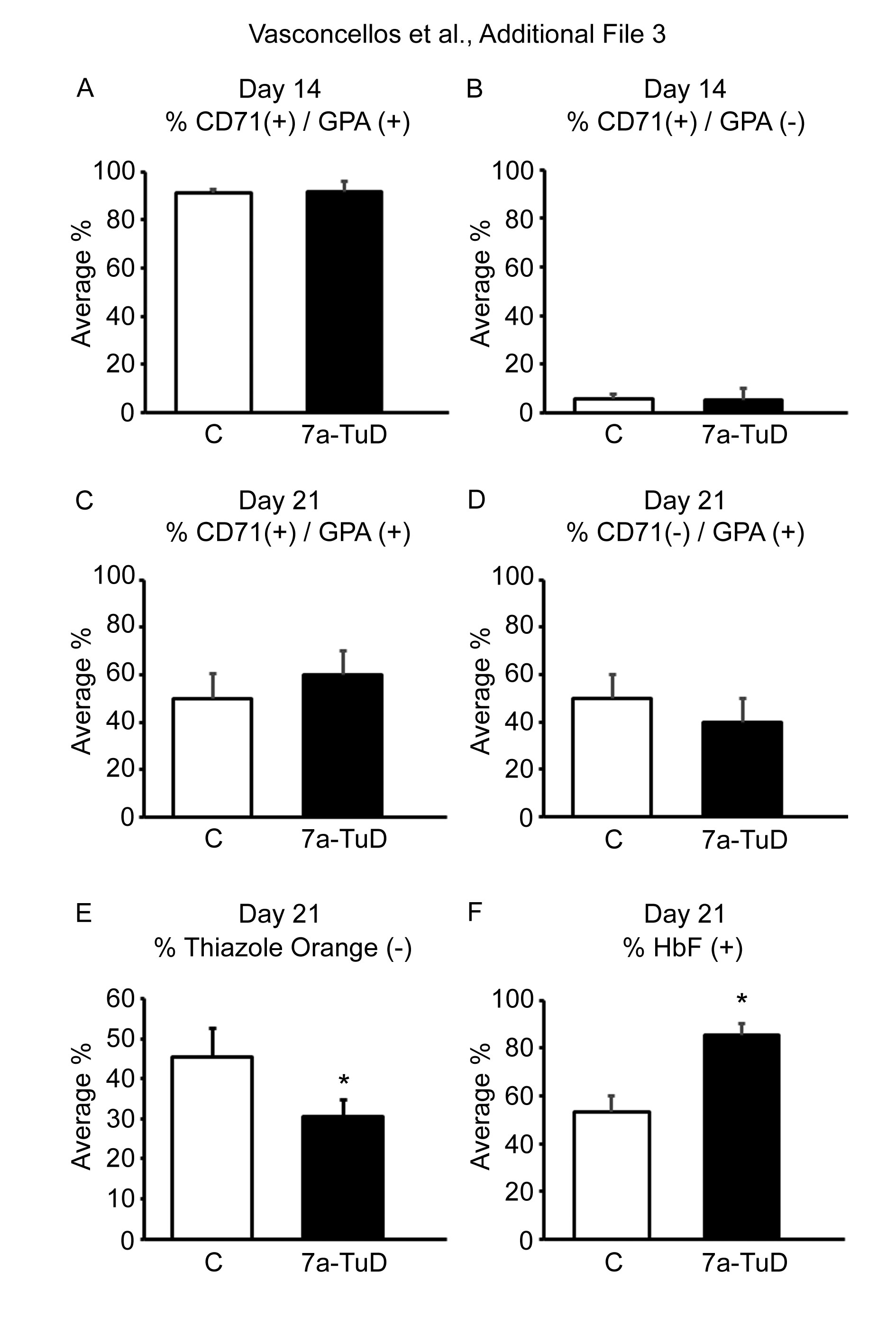

Supplement: Supplementary file 3 — Additional file 3. Quantitative analysis of the flow cytometric results measured by fluorescence-activated cell analysis of control transduction and let-7a-TuD. (A) Percentage of CD71(+) and GPA(+) cells or (B) CD71(+) and GPA(-) cells at culture day 14. (C) Percentage of CD71(+) and GPA(+) cells or (D) CD71(-) and GPA(+) cells at culture day 21. (E) Percentage of thiazole orange negative cells (enucleated) at culture day 21. (F) Percentage of fetal hemoglobin positive cells at culture day 21. Open bars represent control and black bars represent let-7a-TuD. Mean value ± SD of five independent donors for CD71, GPA and thiazole orange stains. Mean value ± SD of three independent donors for HbF stain. P values were calculated using two-tailed Student’s t-test. CD71 = anti-transferrin receptor; GPA = anti-glycophorin A; TO = thiazole orange; HbF = fetal hemoglobin; C = control (negative control vector) transduction; 7a-TuD = let-7a tough decoy design. *p<0.05. [file 12967_2017_1273_MOESM3_ESM.tif]
