# Supplementary material for: Tough decoy targeting of predominant let-7 miRNA species in adult human hematopoietic cells
Source: J Transl Med. 2017 Aug 2;15:169. doi: 10.1186/s12967-017-1273-x (PMC5541688; doi:10.1186/s12967-017-1273-x)
Supplement: Supplementary file 2 — Additional file 2. Evolutionary conservation of the let-7 family of miRNAs from representative species progressing from the worm to human. Mature let-7 sequences from each species compared to the corresponding human sequence. Mature let-7 sequences from all species were obtained from the miRBase database release 21 (http://mirbase.org). Some members of the family may be missing due to incomplete sequencing, rather than their absence from a species. [file 12967_2017_1273_MOESM2_ESM.docx]

**Additional File 2**. Evolutionary conservation of the *let-7* family of miRNAs from representative species progressing from the worm to human. Mature *let-7* sequences from each species compared to the corresponding human sequence. Mature *let-7* sequences from all species were obtained from the miRBase database release 21 (http://mirbase.org). Some members of the family may be missing due to incomplete sequencing, rather than their absence from a species.

| **Species** | ***Let-7* family members** | | | | | | | | |
| --- | --- | --- | --- | --- | --- | --- | --- | --- | --- |
|  | **-*7a*** | **-*7b*** | **-*7c*** | **-*7d*** | **-*7e*** | **-*7f*** | **-*7g*** | **-*7i*** | ***miR-98*** |
| *Ascaris suum* (pigs’ roundworm) |  | X | X | X | X | X | X | X | X |
| *Brugia malayi* (roundworm) |  | X | X | X | X | X | X | X | X |
| *Caenorhabditis brenneri* (small worm) |  | X | X | X | X | X | X | X | X |
| *Caenorhabditis briggsae* (small worm) |  | X | X | X | X | X | X | X | X |
| *Caenorhabditis elegans* (worm) |  | X | X | X | X | X | X | X | X |
| *Caenorhabditis remanei* (worm) |  | X | X | X | X | X | X | X | X |
| *Capitella teleta* (worm) |  | X | X | X | X | X | X | X | X |
| *Pristionchus pacificus* (roundworm) |  | X | X | X | X | X | X | X | X |
| *Ixodes scapularis* (blacklegged tick) |  | X | X | X | X | X | X | X | X |
| *Nasonia giraulti* (jewel wasp) |  | X | X | X | X | X | X | X | X |
| *Nasonia vitripennis* (jewel wasp) |  | X | X | X | X | X | X | X | X |
| *Apis mellifera* (honey bee) |  | X | X | X | X | X | X | X | X |
| *Drosophila ananassae* (fruit fly) |  | X | X | X | X | X | X | X | X |
| *Drosophila erecta* (fruit fly) |  | X | X | X | X | X | X | X | X |
| *Drosophila grimshawi* (fruit fly) |  | X | X | X | X | X | X | X | X |
| *Drosophila melanogaster* (fruit fly) |  | X | X | X | X | X | X | X | X |
| *Drosophila mojavensis* (fruit fly) |  | X | X | X | X | X | X | X | X |
| *Drosophila persimilis* (fruit fly) |  | X | X | X | X | X | X | X | X |
| *Drosophila pseudoobscura* (fruit fly) |  | X | X | X | X | X | X | X | X |
| *Drosophila sechellia* (fruit fly) |  | X | X | X | X | X | X | X | X |
| *Drosophila simulans* (fruit fly) |  | X | X | X | X | X | X | X | X |
| *Drosophila virilis* (fruit fly) |  | X | X | X | X | X | X | X | X |
| *Drosophila willistoni* (fruit fly) |  | X | X | X | X | X | X | X | X |
| *Drosophila yakuba* (fruit fly) |  | X | X | X | X | X | X | X | X |
| *Lottia gigantean* (owl limpet) |  | X | X | X | X | X | X | X | X |
| *Saccoglossus kowalevskii* (acorn worm) |  | X | X | X | X | X | X | X | X |
| *Branchiostoma belcheri* (amphioxus) |  | X | X | X | X | X | X | X | X |
| *Branchiostoma floridae* (lancelet amphioxus) |  | X | X | X | X | X | X | X | X |
| *Petromyzon marinus* (sea lamprey) |  |  |  |  | X | X | X | X | X |
| *Cyprinus carpio* (carp) |  |  | X | X | X | X |  |  | X |
| *Danio rerio* (zebrafish) |  |  |  |  |  |  |  |  | X |
| *Fugu rubripes* (pufferfish) |  |  | X |  |  | X |  |  | X |
| *Ictalurus punctatus* (channel catfish) |  |  |  |  |  |  |  |  | X |
| *Tetraodon nigroviridis* (pufferfish) |  |  | X |  |  | X |  |  | X |
| *Oryzias latipes* (japanese rice fish) |  |  |  | X |  | X |  | X | X |
| *Salmo salar* (Atlantic salmon) |  |  |  |  |  |  |  |  | X |
| *Xenopus tropicalis* (frog) |  |  |  | X |  |  |  |  |  |
| *Anolis carolinensis* (arboreal lizard) |  |  |  |  |  |  |  |  |  |
| *Ophiophagus hannah* (king cobra) |  |  |  |  |  |  |  |  |  |
| *Gallus gallus* (domesticated fowl) |  |  |  |  | X |  |  | X | X |
| *Taeniopygia guttata* (zebra finch) |  |  |  |  |  |  |  |  | X |
| *Sarcophilus harrisii* (tasmanian devil) |  | X | X | X | X | X |  |  | X |
| *Monodelphis domestica* (opossum) |  |  | X |  | X |  |  |  | X |
| *Equus caballus* (horse) |  | X |  |  |  |  |  | X |  |
| *Canis familiaris* (dog) |  |  |  |  |  |  |  | X |  |
| *Sus scrofa* (wild boar) |  | X |  |  |  |  |  |  |  |
| *Ovis aries* (sheep) |  |  |  |  | X |  |  |  | X |
| *Capra hircus* (domestic goat) |  |  |  |  |  |  |  |  |  |
| *Bos Taurus* (taurine cattle) |  |  |  |  |  |  |  |  |  |
| *Rattus norvegicus* (rat) |  |  |  |  |  |  |  |  |  |
| *Mus musculus* (mice) |  |  |  |  |  |  |  |  |  |
| *Gorilla gorilla* (gorilla) |  |  |  | X |  |  |  |  |  |
| *Macaca mulatta* (rhesus monkey) |  |  |  |  |  |  |  |  |  |
| *Pan troglodytes* (chimpanzee) |  |  |  |  |  |  |  |  |  |
| *Pongo pygmaeus* (orangutan) |  |  |  |  |  |  |  |  |  |
| *Homo sapiens* (human) |  |  |  |  |  |  |  |  |  |

Red = perfect match, gray= does not match, X = miRNA not present in the miRBase database.

Additional *let-7* family members present in some species, but not present in humans (example: *let-7h* and *let-7j*) are not listed on the table.
